# Use of in-gel peroxidase assay for cytochrome *c* to visualize mitochondrial complexes III and IV

**DOI:** 10.1242/bio.047936

**Published:** 2020-01-08

**Authors:** Tsukasa Hara, Yuma Shibata, Ryosuke Amagai, Ayako Okado-Matsumoto

**Affiliations:** Department of Biology, Faculty of Science, Toho University, Miyama 2-2-1, Funabashi, Chiba 274-8510, Japan

**Keywords:** Mitochondrial respiratory chain complexes, High-resolution clear native polyacrylamide gel electrophoresis, In-gel activity assay, Supercomplex, Oxidative phosphorylation, Enhanced chemiluminescence

## Abstract

The in-gel activity assay (IGA) is a powerful technique that uses enzymatic activity and compares intensities of detected bands in mitochondrial respiratory chain supercomplexes, and it is applicable to eukaryotic organisms. However, no IGA has been established for complex III because of the difficulty of access by ubiquinol, a substrate for complex III. Herein, we demonstrate that cytochrome *c* (Cyt *c*) showed peroxidase activity on IGA as a component of complexes III and IV. We used pre-incubation with sodium dodecyl sulfate (SDS) before IGA to loosen complexes in the gel after high-resolution clear native polyacrylamide gel electrophoresis (hrCN-PAGE), a refinement of blue native PAGE. The signals of IGA based on peroxidase activity were obtained using enhanced chemiluminescence solution. Then, the gel was directly used in western blotting or hrCN/SDS two-dimensional PAGE. Our findings indicate that IGA for Cyt *c* reflected the indirect activity of complexes III and IV.

## Abstract (precis)

**Summary:** An improved in-gel activity assay visualized respiratory chain complexes III, IV and supercomplexes through cytochrome *c*. Pre-incubation of detergents enhanced the in-gel activity assay.

## INTRODUCTION

The in-gel activity assay (IGA) has been used to detect and simultaneously compare associations of mitochondrial respiratory chain complexes (called ‘supercomplexes’) by semi-quantification of proportional fluctuations in the electron transfer chain (ETC) activity of supercomplexes (Diéguez-Casal et al., 2014; Van Coster et al., 2001; Wittig et al., 2007). IGA is applicable to eukaryotic organisms, e.g. turtle (Bundgaard et al., 2019), bovine, yeast (Ladig et al., 2011), potato (Eubel et al., 2004) and *Drosophila* (Elkholi et al., 2019). Using western blot analysis, it was reported that supercomplex formation was enhanced in genetically adipogenic differentiated human mesenchymal stem cells (Hofmann et al., 2012). Also, it was summarized in a review that deterioration of supercomplex formation modulates cristae morphology and leads to mitochondrial dysfunction (Baker et al., 2019). It has been gradually revealed that supercomplex formation and mitochondrial dynamics are tightly related. Since IGA does not require specific antibodies, it can be a powerful tool for the investigation of supercomplexes in various kinds of eukaryotic organisms as an alternative to western blotting. To date, substrates targeting complexes I, II, IV and V (CI, CII, CIV and CV) have been applicable to IGA-CI, -CII, -CIV and -CV (Wittig et al., 2007), respectively. However, IGA-complex III (IGA-CIII) has not been established because of the difficulty of access by ubiquinol, the substrate of CIII. The diaminobenzidine (DAB) assay, which is commonly used for staining CIII and CIV (Wittig et al., 2007), is based on reduction of cytochrome *c* (Cyt *c*) and oxidation of DAB. Therefore, the DAB assay is suitable for detecting CIV, and when using this assay for detection of CIII, only the CIII that has oxidized Cyt *c* is visualized (Wittig et al., 2007). Recently, it was reported that a tetramethylbenzidine (TMB) solution could be used to stain CIII after blue-native (BN)-PAGE (Smet et al., 2011). TMB solution is normally used for western blotting based on peroxidase activity. Another study reported that an enhanced chemiluminescence (ECL) solution could be used to detect CIII on membranes immediately after protein transfer (Weber-Lotfi et al., 2015). In those reports, bands were only detected in positions with a molecular mass corresponding to CIII, and the authors concluded that the heme core of CIII was involved in peroxidase activity. Improving IGA with ECL solution would require identifying the heme core involved in peroxidase activity and determining why CIV could not be detected with those methods. It was reported that CIII comprises three heme cores (*b*_L_ and *b*_H_ heme in cytochrome *b*, and *c*_1_ heme in cytochrome *c*_1_) and that CIV comprises two heme cores (*a* and *a*_3_ heme in subunit I) as components (Guo et al., 2017; Rich, 2017; Zhang et al., 1998). It was also reported that Cyt *c*, the substrate of CIII and CIV, had peroxidase activity, regardless of whether Cyt *c* is in reduced form or oxidized form (Radi et al., 1991; Yin et al., 2017). Therefore, Cyt *c* may mainly react with TMB and ECL solution.

In this study, we applied ECL solution for IGA to detect peroxidase activity exhibited from CIII and CIV by pre-incubation with detergents. Then, we investigated whether Cyt *c* was responsible for the peroxidase activity of CIII and CIV by high-resolution clear-native polyacrylamide gel electrophoresis (hrCN-PAGE). hrCN-PAGE is a refinement of BN-PAGE (Wittig et al., 2007) in which one anionic detergent, such as deoxycholic acid (DOC), and one or more neutral detergent(s), such as *n*-dodecyl-*β*-D-maltoside (DDM), are added in the cathode buffer instead of CBB G-250 of BN-PAGE. The mixed micelles formed by the detergents confer to the proteins the net negative charge necessary for their solubilization and migration to the anode, allowing the separation of complexes with a high resolution.

## RESULTS AND DISCUSSION

### Improving the sensitivity of in-gel peroxidase activity assay

**Fig. 1.**
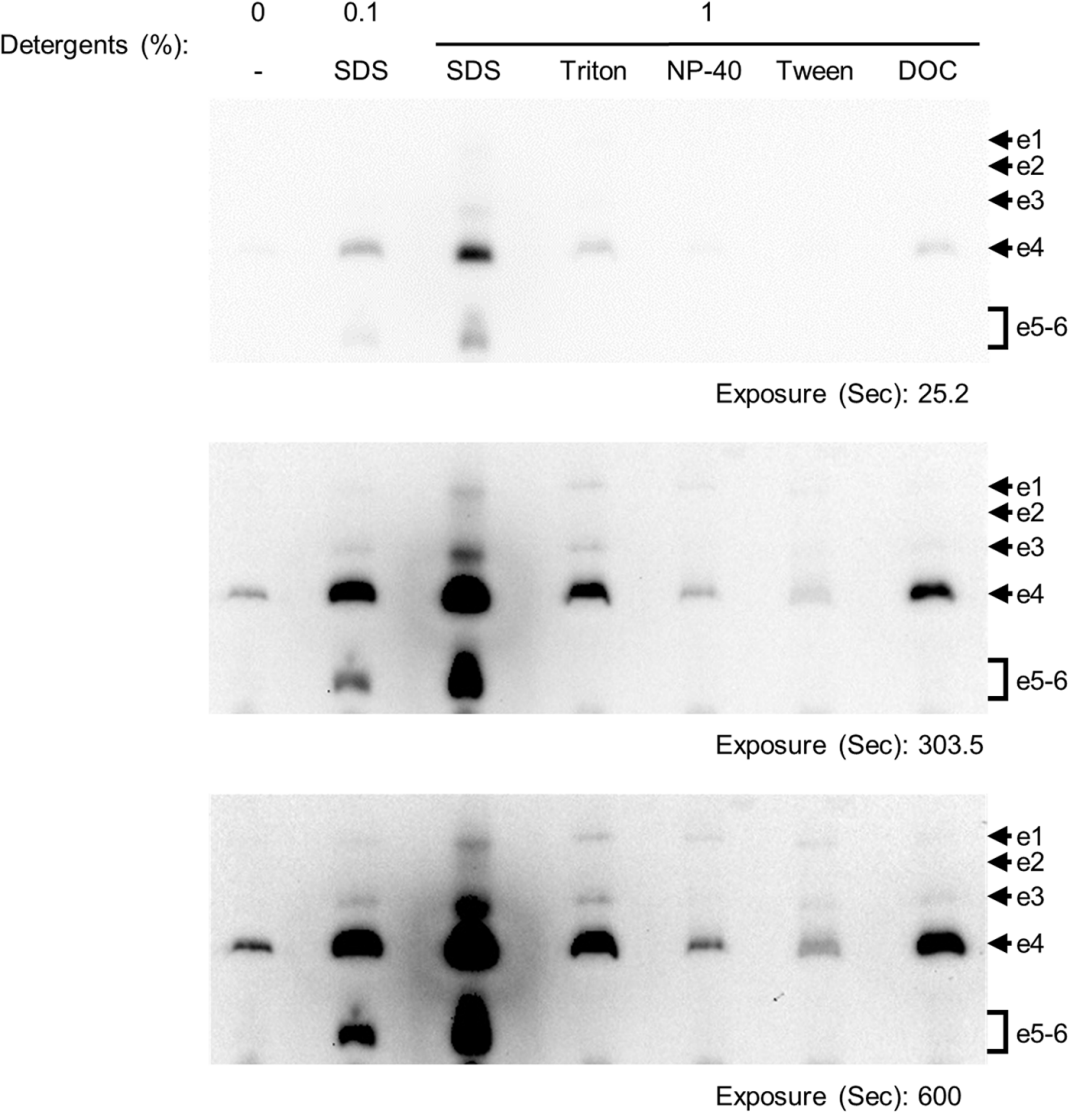
**Enhancement of in-gel peroxidase assay by detergents.** 20 μg/lane of membrane protein was separated by hrCN-PAGE. Peroxidase activity was assayed with ECL solution after pre-incubation with detergents. The signals of exposure times at 25.2, 303.5 and 600 s are shown. The signals e1–e6 are identical to Fig. 2. NP-40, NP-40 substitute; SDS, sodium dodecyl sulfate; Triton, Triton X-100; Tween, Tween 20; DOC, deoxycholic acid.

The effect of detergents on IGA, based on peroxidase activity, was investigated (Fig. 1). 1% sodium dodecyl sulfate (SDS) yielded the greatest enhancement of signals of peroxidase activity. This was not surprising considering that peroxidase activity was likely exhibited by Cyt *c*, because SDS was previously reported to enhance the peroxidase activity of Cyt *c* by spectrophotometry (Vladimirov et al., 2006). Since the heme core of Cyt *c* is tightly enclosed in the peptide frame, SDS opens the frame and thus might increase peroxidase activity. Therefore, Cyt *c* should exhibit peroxidase activity. The detergents NP-40 substitute and Tween 20 enhanced signals e1 and e3, and Triton X-100 and DOC enhanced signals e1, e3 and e4. This suggests that those detergents were milder than SDS and, therefore, could enhance only signals of Cyt *c*, a component of CIII. Among the four detergents, Triton X-100 yielded the greatest enhancement of signals for peroxidase activity (Fig. 1). DOC is an anionic detergent and therefore likely has the same effect as SDS. Intriguingly, Triton X-100 is a nonionic detergent that solubilizes the mitochondrial membrane for extraction of supercomplexes (Schägger and Pfeiffer, 2000), and it was fortunate that enhancement of IGA only at positions corresponding to the molecular mass of CIII was advantageous in analyzing the indirect activity of CIII.

**Fig. 2.**
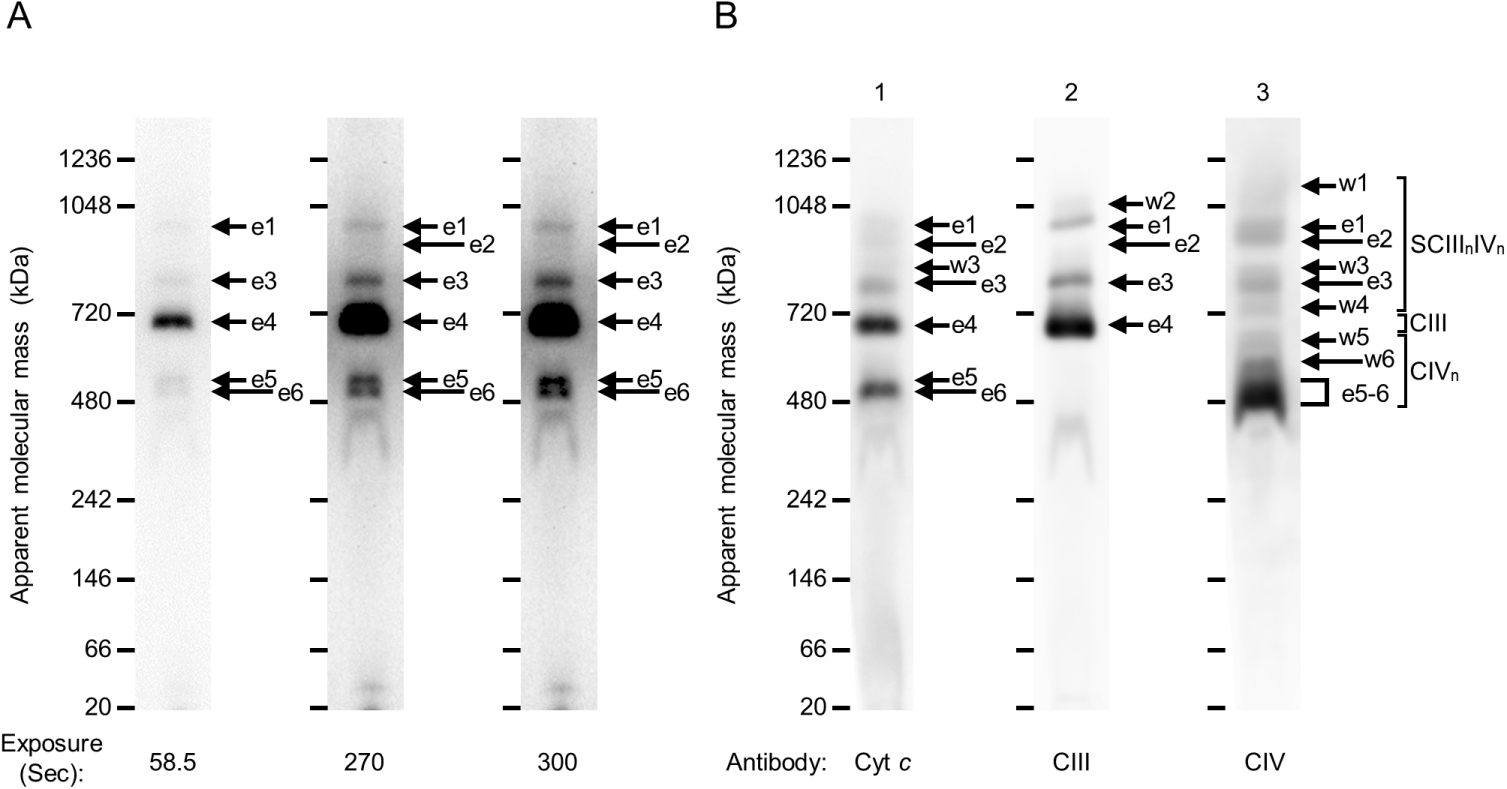
**Direct comparison of signals obtained after in-gel peroxidase activity assay and western blotting.** 20 μg/lane of membrane protein was separated by hrCN-PAGE in a 3–14% gradient gel. (A) Peroxidase activity was assayed by the ECL solution after pre-incubation of 1% SDS solution. Exposure time of 58.5, 270 and 300 s are shown. e1–e6 denote signals detected by the ECL solution and western blotting. (B) Cyt *c* (anti-Cyt *c,* lane 1), CIII (anti-Core I, lane 2) and CIV (anti-COX I, lane 3) were detected by western blotting with exposure times of 600, 60 and 40 s, from left to right. e1–e6 are shown as signals detected at the same position with the molecular mass of Fig. 1A. w1–w6 denote signals detected by western blotting. Cyt *c*, cytochrome *c*; CIII, complex III; CIV, complex IV; IGA, in-gel activity assay; SCIII_n_IV_n_, supercomplex III_n_IV_n_.

### Direct comparison with signals of IGA and western blotting

**Fig. 3.**
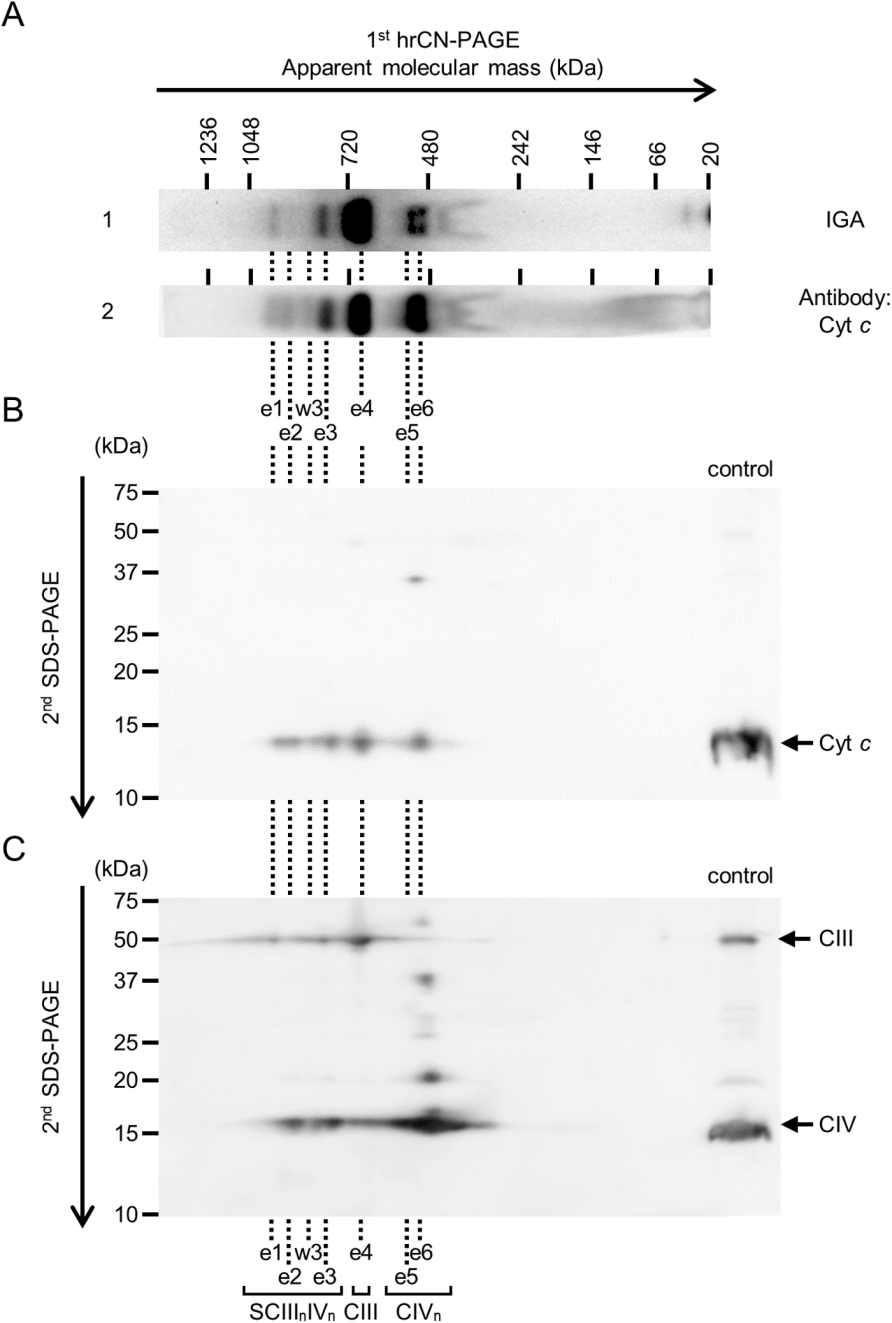
**Association of Cyt c with CIII and CIV revealed by hrCN/SDS 2D-PAGE**. 40 μg of the membrane protein was separated by 1D hrCN-PAGE. Then, a gel strip of first dimensional hrCN-PAGE was soaked in denaturation solution and alkylation solution and the gel strip was set on the top of second-dimensional SDS-PAGE. (A) Although the Cyt *c* signals on the first dimensional hrCN-PAGE were not visualized, the Cyt *c* signals visualized by in-gel peroxidase assay (lane 1) and western blotting with anti-Cyt *c* (lane 2) are shown for easy comparison, using the same images shown in Fig. 2A and B-1. (B) Cyt *c* was visualized by western blotting (anti-Cyt *c*) with an exposure time of 110 s. (C) Repeatedly using the same membrane of Cyt *c*, CIII (anti-Core I) and CIV (anti-COX IV) were detected with an exposure time of 110 s. The signals e1–e6 and w3 are identical to Fig. 2. Cyt *c*, cytochrome *c*; CIII, complex III; CIV, complex IV; IGA, in-gel activity assay; SCIII_n_IV_n_, supercomplex III_n_IV_n_.

To identify the heme core involved in peroxidase activity and determine why CIV could not be detected with ECL solution in a previous report (Weber-Lotfi et al., 2015), rat heart mitochondrial membrane proteins were separated by hrCN-PAGE, and a gel of three lanes was incubated in 1% SDS. Then, CIII and CIV_n_ were visualized with ECL solution (Fig. 2A). After visualization, proteins in the gel were transferred onto a PVDF membrane, after which the membrane was cut and separated into three strips for the detection of Cyt *c*, CIII and CIV (Fig. 2B). These results indicate that an ECL solution can be applied to detect CIII and CIV_n_ by pre-incubation with SDS in a gel. A considerable advantage of this method is that after treatment with ECL solution, the gel can be directly used for western blotting or 2D electrophoresis, which allows for direct comparison with western blot signals. On western blotting, some signals of CIII and CIV_n_ were detected on separate membranes at the same positions of molecular mass, such as signals e1, e2 and e3 (Fig. 2B-2,B-3). Therefore, e1, e2 and e3 were likely associated as supercomplexes of CIII and CIV (SCIII_n_IV_n_). In a comparison of IGA signals using ECL solution and western blot signals, all IGA signals from e1 to e6 were matched to western blot signals targeting Cyt *c,* while IGA signals were partially matched to western blot signals targeting CIII and CIV (Fig. 2)*.* Furthermore, some additional signals, w1 to w6, were detected by western blotting (Fig. 2B). Cyt *c* signals of the first dimensional hrCN-PAGE were separated by the second dimensional SDS-PAGE (Fig. 3). Since Cyt *c* signals of hrCN-PAGE contained CIII and/or CIV, our results indicate that Cyt *c* was associated with CIII and/or CIV. Previous studies suggested that heme cores of CIII exhibited peroxidase activity, and signals equivalent to e1, e3 and e4 were detected by TMB solution (Smet et al., 2011) or ECL solution (Weber-Lotfi et al., 2015), but signals equivalent to e2, e5 and e6 were not detected. In this study, hrCN-PAGE gel was pre-incubated with SDS before IGA to loosen complexes, and signals containing CIV_n_ were also detected (Fig. 2A). Cyt *c*, CIII and CIV have heme cores (Guo et al., 2017; Radi et al., 1991; Rich, 2017; Yin et al., 2017; Zhang et al., 1998), and they are capable of exhibiting peroxidase activity. We revealed that ECL solution reacts with Cyt *c*. Flexible association of Cyt *c* with CIII and CIV suggests the plasticity of the complicated environment around supercomplexes. Superoxide dismutase 2 (SOD2) (Suthammarak et al., 2013), some acyl-CoA dehydrogenases (VLCAD, LCAD and MCAD), mitochondrial trifunctional protein (TFP), electron transfer flavoprotein (ETF) (Wang et al., 2010) and optic atrophy 1 protein (OPA1) (Zanna et al., 2008) are reported to be associated with supercomplexes, which suggests that the supercomplex environment is more complicated than previously indicated.

There have been conflicting discussions on the supercomplex formation and its electron flux. Acin-Perez et al. revealed that the supercomplex cut out from a BN-PAGE gel consumed oxygen with nicotinamide adenine dinucleotide as a substrate for CI, and the oxygen consumption was inhibited by potassium cyanide as an inhibitor for CIV. Therefore, they suggested that Cyt *c*, which was contained in supercomplex, played the role of a functional structure to transfer electrons (Acín-Pérez et al., 2008). Recently, the architecture of mitochondrial respiratory megacomplex I_2_III_2_IV_2_ was analyzed by cryo-electron microscopy, and a gap between the accepting site of Cyt *c* between CIV and CIII was calculated around 10–11 nm from one CIV to the other two CIIIs. Since the diameter of Cyt *c* was around 3 nm, they suggested that an electron transfer between CIII and CIV would be based on diffusion (Guo et al., 2017). Trouillard et al. also suggested that an electron transfer from CIII to CIV was mainly based on the Cyt *c* diffusion. They revealed that 16% of Cyt *c* showed rapid electron transfer and the other showed a diffusion-like reaction by monitoring the photoactivated reactions of CIV in intact yeast (Trouillard et al., 2011). They concluded that this rapid electron transfer was due to pre-bound Cyt *c* to CIV, and not due to an effective trapping of the soluble carrier within the supercomplex. The function of supercomplexes as respirasomes has not been fully elucidated, and nor has the distance of accepting site Cyt *c* between CIII and CIV. We demonstrated that the effects of pre-incubation with different kinds of detergents on in-gel peroxidase assay were varied (Fig. 1). NP-40 substitute and Tween 20 loosened the structure of supercomplexes (SCIII_n_IV_n_), which were detected as signals of e1 and e3. However, those detergents did not loosen an association between Cyt *c* and CIII or CIV_n_, which were detected as signals e4 and e5–6, respectively. Likewise, Triton X-100 and DOC loosened both structures of SCIII_n_IV_n_ and CIII, which were detected as signals e1, e3 and e4. In our study, SDS loosened all structures that were included in the signal e2 (SCIII_n_IV_n_)_._ Therefore, further analysis of IGA were applied with pre-incubation in 1% SDS solution. Furthermore, we demonstrated that the components of complexes such as SCIII_n_IV_n_, Cyt *c*, CIII and CIV_n_ were dissociated by hrCN/SDS two-dimensional PAGE (hrCN/SDS 2D-PAGE) (Fig. 3). Our results support the report that the functional differences of supercomplexes are based on membrane-solubilized detergents such as DDM or digitonin (Acín-Pérez et al., 2008). Consequently, use of ECL solution in an IGA for mitochondrial membranes is better referred to as IGA-Cyt *c* rather than IGA-CIII, as in previous reports (Smet et al., 2011; Weber-Lotfi et al., 2015).

**Fig. 4.**
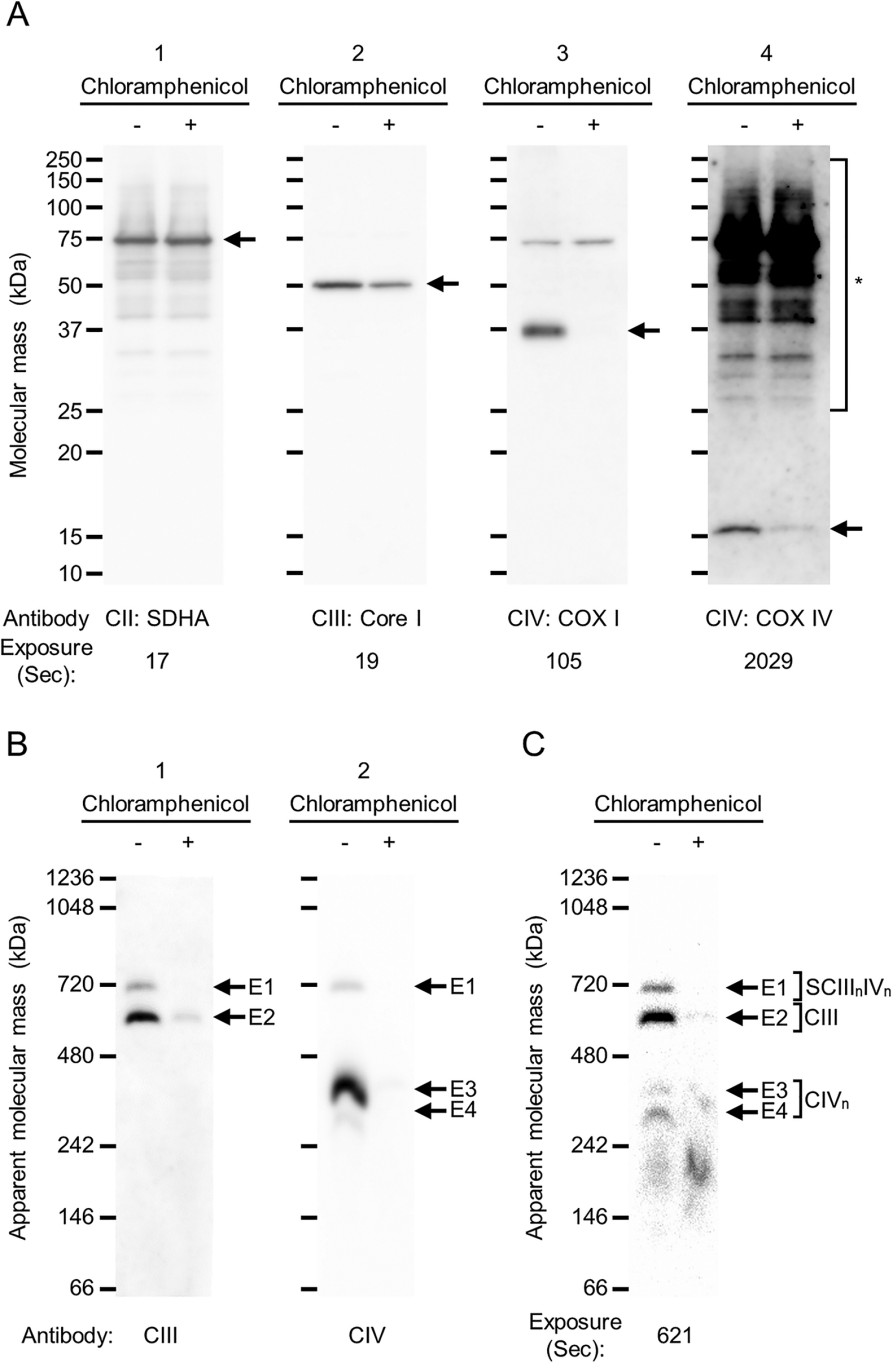
**Depletion of mitochondrially encoded components of CIII and CIV in N2a cells by inhibitor of mitochondrial translation**. Mouse neuroblastoma N2a cells were treated for 120 h with chloramphenicol, which is an inhibitor of mitochondrial translation. (A) The effects of chloramphenicol treatment for the protein synthesis of components were examined. 5 μg/lane of whole-membrane protein of N2a cells with (+) or without (−) chloramphenicol treatment was separated by SDS-PAGE, and the components of complexes II, III and IV were detected by western blotting using anti-SDHA (A-1), anti-Core I (A-2) and anti-COX I antibodies (A-3). Then, anti-COX IV was reprobed onto the anti-SDHA-probed membrane without the stripping process, and complex IV was detected (A-4). Each arrow indicates SDHA (A-1), Core I (A-2), COX I (A-3) and COX IV (A-4). Asterisk indicates unstripping SDHA signals on the PVDF membrane (A-4). (B,C) 15 μg/lane of whole-membrane protein of N2a cells with (+) or without (−) chloramphenicol treatment were separated by hrCN-PAGE in a 3–14% gradient gel. E1–E4 denote signals detected by western blotting (B) and ECL solution (C). CIII (anti-Core I, B-1) and CIV (anti-COX I, B-2) were detected by western blotting with exposure times of 58 and 17 s, respectively. E1–E4 are shown as signals detected at the same position with the molecular mass. Peroxidase activity was assayed by the ECL solution after pre-incubation of 1% of SDS solution (C). CII, complex II; CIII, complex III; CIV, complex IV; SCIIInIVn, supercomplex IIInIVn.

The specificity of IGA-Cyt *c* for the CIII and CIV assay was confirmed by further experiments. Since mitochondrial respiratory chain CIII and CIV were encoded both in the mitochondrial and nuclear DNA (Fernández-Vizarra and Zeviani, 2015; Mick et al., 2011), the treatment with chloramphenicol, which is an inhibitor of mitochondrial translation, led to the depletion of fully assembled CIII and CIV in mouse neuroblastoma N2a cells. Firstly, the protein expression of the components of CII, CIII and CIV were validated by SDS-PAGE and western blotting using antibodies targeting succinate dehydrogenase subunit A (SDHA) for CII, ubiquinol-cytochrome *c* reductase core protein I (Core I) for CIII and cytochrome *c* oxidase subunit I and IV (COX I and COX IV) for CIV (Fig. 4A). Since all components of CII were encoded in the nuclear DNA, SDHA protein was stably expressed in chloramphenicol-treated N2a cells (Fig. 4A). Concerning the protein expression of CIV, mitochondrially-encoded COX I was completely depleted in chloramphenicol-treated N2a cells, and nuclear-encoded COX IV protein was also downregulated by chloramphenicol treatment. Similarly, nuclear-encoded Core I protein, which is a component of CIII, was also downregulated (Fig. 4A). Our results coincided with Konovalova's report that nuclear-encoded components of CI (NDUFA9: NADH ubiquinone oxidoreductase subunit A9) and CIII (Core II) were also downregulated in chloramphenicol-treated human neuroblastoma SH-SY5Y cells (Konovalova, 2019). Therefore, depletion of mitochondrially encoded components could affect other protein expressions of nuclear-encoded components of respiratory chain complexes. The assembly of respiratory chain complexes was then examined by hrCN-PAGE and western blotting using anti-Core I antibody for CIII and anti-COX I antibody for CIV. The signals E1 (SCIII_n_IV_n_), E2 (CIII), E3 and E4 (CIV_n_) were detected in N2a cells, while only E2 was detected in chloramphenicol-treated N2a cells (Fig. 4B). Similarly, E2 signal was barely detectable by IGA-Cyt *c* (Fig. 4C). Therefore, our results suggest that IGA-Cyt *c* was detected by the peroxidase activity of Cyt *c* in CIII and CIV, and the sensitivity of IGA-Cyt *c* was comparable to the western blotting of CIII. Furthermore, the sensitivity of IGA-Cyt *c* for SCIII_n_IV_n_, CIII, and CIV_n_ was improved by the pre-incubation with SDS.

Finally, we confirmed IGA-Cyt *c* with treatment by SDS in over 10 independent experiments and tested IGA-Cyt *c* using six other rat organs (spinal cord, brain, lung, liver, spleen and kidney) and human leukocytes, which have been examined IGA-CI (Hara et al., 2016) and IGA-Cyt *c*, with similar results (data not shown).

### Conclusions

Using an in-gel peroxidase activity assay, dubbed IGA-Cyt *c*, we demonstrated that Cyt *c* was visualized in gel as a flexible component of CIII and CIV. Cyt *c* was identified by hrCN/SDS 2D-PAGE as signals detected at the same positions of molecular mass. While the association between CIV and Cyt *c* was unchanged by pre-incubation of the gel after electrophoresis with non-ionic detergents such as Triton X-100, NP-40 substitute and Tween 20, the association between CIII and Cyt *c* was altered by these detergents, which allowed us to distinguish Cyt *c* associated with CIII and CIV. Theoretically, the transient presence of Cyt *c* reflects the respiration balance of ETC. By comparing the intensity of IGA-Cyt *c* with western blot signals for CIII, the indirect activity of CIII could be calculated among samples obtained from different groups. Although Triton X-100 and DOC were useful for visualizing Cyt *c* associated with CIII, gel strips should be pre-incubated with SDS to visualize all Cyt *c* associated with CIII and CIV, including SCIII_n_IV_n_. This method can be applied to various kinds of eukaryotic organisms.

## MATERIALS AND METHODS

### Animals

Wistar rats (*Rattus norvegicus*, 8-week-old males) were purchased from CLEA Japan, Inc. (Japan). All the animal experiments were conducted in compliance with a protocol reviewed and approved by the Toho University Animal Care and User Committee (17-53-292).

### Cell culture and inhibition of mitochondrial translation

Mouse neuroblastoma N2a cells (Okado-Matsumoto and Fridovich, 2002) were cultured in Dulbecco's Modified Eagle's Medium (DMEM high-glucose, Nacalai Tesque, Japan) in a humidified cell incubator at 37°C in a 5% CO_2_ atmosphere. The medium was supplemented with 10% (v/v) fetal bovine serum (FBS; Biosera, France) and 1% (v/v) Penicillin-Streptomycin Mixed Solution (Nacalai Tesque).

Mitochondrial translation was inhibited in N2a cells by treatment with chloramphenicol (Nacalai Tesque) (Sun et al., 2016). 2×10^6^ cells were cultured in tissue culture dishes (100×20 mm, Thermo Fisher Scientific). After 18 h of cell culture, 40 μg/ml chloramphenicol was added into the culture medium, and the cells were incubated for 120 h. The chloramphenicol-treated culture medium was changed every 24 h. Then the chloramphenicol-treated cells were harvested and washed three times with phosphate-buffered saline, and stored at −80°C until use.

### Isolation of mitochondria

Fresh Wistar rat hearts were minced with knives, then homogenized with a Dounce homogenizer (Wheaton, USA) at 50 strokes with four times the volume of sucrose buffer [10 mM Tris-HCl (pH 7.4), 250 mM sucrose and 1 mM EDTA] on ice. The supernatant after centrifugation at 1000× ***g*** for 10 min at 4°C was then pelleted by centrifugation at 20,000× ***g*** for 20 min at 4°C. The pellet was washed three times with sucrose buffer and stored at −80°C until use.

### Solubilization of membranes

After three freeze–thaw cycles with 50 mM phosphate buffer (pH 7.4), rat heart mitochondrial membranes and whole-cell membranes of N2a were pelleted by ultracentrifugation (Beckman Coulter, USA) at 100,000× ***g*** for 60 min at 4°C. Then, the pellet was washed twice with 50 mM phosphate buffer by ultracentrifugation at 100,000× ***g*** for 60 min at 4°C and stored at −80°C until use. The 5-mg wet mitochondrial membrane pellet of rat heart was solubilized with 100 μl of membrane lysis buffer [MLB; Bis-Tris (pH 7.0), 50 mM sodium chloride and 500 mM *ε*-aminocaproic acid] in the presence of digitonin and mixed in a tube mixer at maximum speed for 30 min at 4°C (MT-360; TOMY, Japan). The digitonin-to-wet membrane weight ratio (g/g) in MLB was 1 g/g. Whole-cell membranes of N2a cells were solubilized with digitonin and DDM in the presence of 0.25 g/g and 0.01 g/g, respectively. Then, the membrane fractions were ultracentrifuged at 100,000× ***g*** for 30 min at 4°C, and the supernatants were transferred to other microtubes for the solubilized membrane samples and stored at −80°C until use. Each protein concentration was determined by the Bradford method (Bradford protein assay dye reagent concentration, Bio-Rad, USA), with bovine serum albumin (WAKO, Japan) as the standard.

### hrCN-PAGE and hrCN/SDS 2D-PAGE

A previously described method (Wittig et al., 2007), with minor modifications (Hara et al., 2016), was used to separate respiratory chain complexes by hrCN-PAGE. For making gradient gels, 3% and 14% acrylamide gel solutions [each concentration of 32:1 acrylamide/bis-acrylamide, 50 mM Bis-Tris (pH 7.0), 500 mM *ε*-aminocaproic acid and 20% (v/v) glycerol, which was added only for the 14% gel solution] were prepared. Gradient gels with a thickness of 1 mm were made with a gradient former (Model 485; Bio-Rad) and a Mini-PROTEAN 3 multicasting chamber (Bio-Rad). The anode buffer was prepared with NativePAGE running buffer (Thermo Fisher Scientific), and the cathode buffer contained 0.05% DOC and 0.01% DDM in anode buffer. NativeMark (Thermo Fisher Scientific) was used as the mobility marker, to indicate apparent molecular mass.

For hrCN/SDS 2D-PAGE, 40 μg of the sample was separately applied by 20 μg each to two adjacent lane of a 3–14% gel. After first-dimensional hrCN-PAGE, the gel strip was then treated with two solutions for denaturation and alkylation at 37°C. In both steps, 2× SDS-PAGE sample buffer [62.5 mM Tris-HCl (pH 6.8), 2% SDS, 25% glycerol and 0.01% Bromophenol Blue], which was normally used with reductant and mixed with same volume of samples for sample preparation, was used with additional reagents. For denaturation, the gel strip was incubated with 2× SDS-PAGE sample buffer including 50 mM 2-mercaptoethanol for 30 min. Then, for alkylation, the solution was replaced with 2× SDS-PAGE sample buffer containing 50 mM *N,N*-dimethylacrylamide for 15 min. The gel strip was then set on the top of second-dimensional SDS-PAGE gel (4% for stacking gel and 15% for separation gel) and sealed in place with 0.5% agarose. For the running control of 2D-PAGE, 5 μg of sample, which was same sample used for the 1D hrCN-PAGE, was applied.

### IGA

CIII and CIV were visualized based on peroxidase activity (Smet et al., 2011; Weber-Lotfi et al., 2015), with modifications in order for the gel to be used directly in western blotting after this IGA. For our study, gel strips were soaked for pre-incubation with Milli-Q water (Merck, Germany), 0.1% or 1% SDS or 1% Triton X-100, NP-40 substitute (WAKO, Japan), Tween 20 or DOC solution for 20 min at room temperature. Solutions were then replaced with Milli-Q water to wash the gel strips for 10 min. Then, the gels were placed on the stage of a ChemiDoc XRS+ system (Bio-Rad), after which ECL solution (EzWestLumi plus, ATTO, Japan) was poured on the gels. Signals of complexes were obtained with the method used for the normal chemiluminescence reaction.

### Western blotting

For western blotting, gels after visualization by ECL solution were incubated for 30 min at 37°C with denaturing buffer [25 mM Tris-HCl (pH 8.3), 192 mM glycine, 0.1% SDS and 5 mM 2-mercaptoethanol]. Proteins in gels were then transferred onto an Immun-Blot PVDF Membrane (Bio-Rad) with transfer buffer [25 mM Tris-HCl (pH 8.3), 192 mM glycine and 0.01% SDS] by using a Mini Trans-Blot electrophoretic transfer cell (Bio-Rad). The membrane was blocked by treatment for 60 min with 5% skim milk diluted in 50 mM Tris-HCl (pH 7.4), 135 mM NaCl and 0.05% Tween 20.

CII, CIII, CIV and Cyt *c*, were detected by using the following antibodies: 1:5000 diluted anti-SDHA (clone: 2E3GC12FB2AE2, 459200, Thermo Fisher Scientific) for CII, 1:5000 diluted anti-Core I (clone: 16D10AD9AH5, ab110252, Abcam, UK) for CIII, 1:3000 diluted anti-COX IV (clone: 20E8C12, ab14744, Abcam) for CIV, 1:5000 diluted anti-COX I (clone: 1D6E1A8, 459600, Thermo Fisher Scientific) for CIV, and 1:5000 diluted anti-cytochrome *c* (clone: A-8, sc-13156, Santa Cruz Biotechnology, USA). All subunits of CII, Core I, which is a subunit of CIII, and COX IV, which is a subunit of CIV, are encoded by the nuclear DNA and synthesized on cytoplasmic ribosomes, and then imported into mitochondria (Fernández-Vizarra and Zeviani, 2015; Kita et al., 1990; Mick et al., 2011). COX I, which is a component of CIV, is encoded by the mitochondrial DNA and synthesized on mitochondrial ribosomes (Mick et al., 2011). All antibodies were diluted in Can Get Signal Immunoreaction Enhancer Solution (TOYOBO, Japan). EzWestLumi plus (ATTO, Japan) was used for horseradish peroxidase detection. The signal was detected with the ChemiDoc XRS+ system (Bio-Rad) or ImageQuant LAS 4010 (GE Healthcare, UK).
